# A Novel c.554+5C>T Mutation in the DUOXA2 Gene Combined with p.R885Q Mutation in the DUOX2 Gene Causing Congenital Hypothyroidism

**DOI:** 10.4274/jcrpe.2380

**Published:** 2016-06-06

**Authors:** Xiao Zheng, Shao-Gang Ma, Ya-Li Qiu, Man-Li Guo, Xiao-Juan Shao

**Affiliations:** 1 Huai’an Hospital Affiliated to Xuzhou Medical College and Huai’an Second People’s Hospital, Clinic of Endocrinology and Metabolism, Huai’an, China; 2 Women and Children’s Hospital of Suqian, Clinic of Neonatal Screening and Care, Suqian, China

**Keywords:** congenital hypothyroidism, dual oxidase maturation factor 2, dual oxidase 2, mutation

## Abstract

The coexistence of mutations in the dual oxidase maturation factor 2 (DUOXA2) and dual oxidase 2 (DUOX2) genes is rarely identified in congenital hypothyroidism (CH). This study reports a boy with CH due to a novel splice-site mutation in the DUOXA2 gene and a missense mutation in the DUOX2 gene. A four-year-old boy was diagnosed with CH at neonatal screening and was enrolled in this study. The DUOXA2, DUOX2, thyroid peroxidase (TPO), and thyrotropin receptor (TSHR) genes were considered for genetic defects screening. Genomic DNA was extracted from peripheral blood leukocytes, and Sanger sequencing was used to screen the mutations in the exon fragments. Family members of the patient and the controls were also enrolled and evaluated. The boy harbored compound heterozygous mutations including a novel splice-site mutation c.554+5C>T in the maternal DUOXA2 allele and c.2654G>A (p.R885Q) in the paternal DUOX2 allele. The germline mutations from his parents were consistent with an autosomal recessive inheritance pattern. No mutations in the TPO and TSHR genes were detected. A novel splice-site mutation c.554+5C>T in the DUOXA2 gene and a mutation p.R885Q in the DUOX2 gene were identified in a 4-year-old patient with goitrous CH.

## WHAT IS ALREADY KNOWN ON THIS TOPIC?

The dual oxidase maturation factor 2 (DUOXA2) and dual oxidase 2 (DUOX2) genes are rarely identified in congenital hypothyroidism (CH).

## WHAT THIS STUDY ADDS?

We detected a novel splice-site mutation in the DUOXA2 gene and a missense mutation in the DUOX2 gene in a boy with CH.

## INTRODUCTION

It is generally known that congenital hypothyroidism (CH) is the most common neonatal endocrine disorder and occurs in approximately 1:2000-1:4000 of newborns. CH cases are caused by various defects including thyroid dysgenesis and thyroid hormone synthesis defects (1,2). Previous studies revealed inactivating mutations in a specific subtype of CH. Of the known genes, mutations in dual oxidase maturation factor 2 (DUOXA2), dual oxidase 2 (DUOX2), thyroid peroxidase (TPO), and thyrotropin receptor (TSHR), that are all known to be related to thyroid dysgenesis or dyshormonogenesis and that are all inherited in an autosomal recessive pattern, have been reported (3,4,5,6).

The existing data suggest that inactivating mutations in the TSHR gene are responsible for thyrotropin (TSH)resistance and thyroid dysgenesis (1,2,6,7). Mutations in the DUOXA2, DUOX2, and TPO genes are responsible for thyroid dyshormonogenesis and goitrous congenital hypothyroidism (GCH) (3,4,5). Defective thyroid hormone synthesis represents most cases of GCH. Mutations in the DUOXA2, DUOX2, TPO, and TSHR genes are more common than those in thyroglobulin (TG) and paired box 8 (PAX8) genes in CH (1,2).

Currently, it is believed that H2O2 generation needs the catalytic core of DUOX2. Oxidation reaction is crucial for the iodination of TG during thyroid hormone synthesis. DUOXA2 is required for normal DUOX2 enzymatic activity. It has been identified that DUOXA2 is crucial for DUOX2 maturation, and genetic defects in DUOX2 cause CH and subclinical another important candidate gene for CH and SCH (3,4,8,9,10).

To date, the genetic defects in CH have not been fully understood. In this study, the DUOXA2, DUOX2, TPO, and TSHR genes were considered for screening genetic defects in a male patient with GCH reported below.

## CASE REPORT

A four-year-old boy came from the city of Suqian in Jiangsu Province, China. He had been diagnosed as CH at the neonatal screening and treatment was initiated. He was recruited by our team for investigation of a possible mutation. The patient was born to non-consanguineous parents without thyroid disease. CH was diagnosed on the basis of serum TSH, free thyroxine (fT4), and free triiodothyronine (fT3) levels. Daily L-thyroxine was administered to the patient at diagnosis. Thyroid gland examinations were performed with 99mTc thyroid scan and ultrasound at age four years. A total of 105 unrelated healthy controls were enrolled in this study. This study was approved by the ethics committee of the hospital. Written informed consent was obtained. Blood samples were collected from the participants.

 At the beginning of the study, venous blood samples were obtained from the boy. DNA was extracted from peripheral blood leukocytes. Primers were designed to target the flanking intron regions of the exons. All exons of the DUOXA2 (MIM# 612772, GenBank NM_207581.3), DUOX2 (MIM# 606759, GenBank NM_014080.4), TPO (MIM# 606765, GenBank NM_000547.5), and TSHR (MIM# 603372, GenBank NM_000369.2) genes were amplified by polymerase chain reaction (PCR). The amplified PCR products were Sanger sequenced directly for variance analysis. All exons of the above genes were first amplified in the patient. If a mutation was identified, the target fragment was also amplified in the patient’s parents and in 105 control individuals. Novel mutations were analyzed by bioinformatic tools.

**Figure 1:**
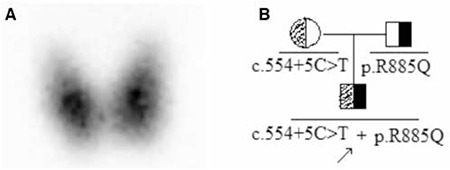
Thyroid 99mTc scan revealed the enlarged thyroid lobes of the boy (Panel A, anterior view). The arrow indicating the proband in the pedigree with the compound heterozygous mutations (Panel B)

The clinical summary and thyroid function of the boy and his parents are shown in Table 1. The proband had overt CH at neonatal screening. L-thyroxine was the treatment of choice at diagnosis, with a starting dose of 10 μ was the treatment of choice at diagnosis, with a starting dose of 10s are shown in Table 1. The proband had. Thyroid function tests showed that the parents had normal thyroid function. Thyroid ultrasound examination demonstrated enlarged thyroid lobes in our patient. Thyroid 99mTc scan revealed that the boy’s thyroid appeared normally located but enlarged (Figure 1, Panel A).

**Figure 2:**
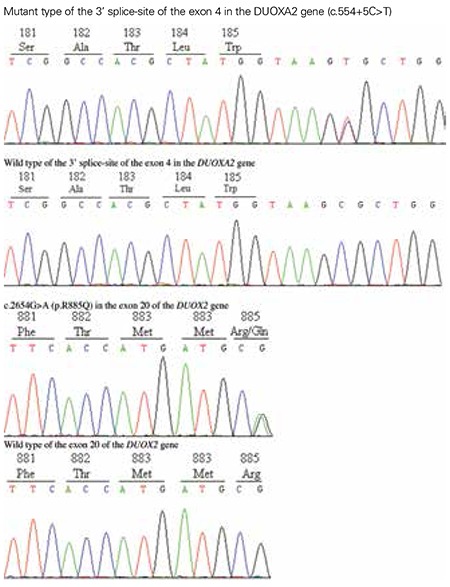
The genotypes revealing the heterozygous mutations in the DUOXA2 gene (c.554+5C>T) and in the DUOX2 gene (c.2654G>A, p.R885Q)

 As shown in Figure 2, the genetic analysis demonstrated two heterozygous mutations, a novel maternal allele splicing site variant (c.554+5C>T) (C to T substitution at position +5 of the donor site of intron 4) in the DUOXA2 gene and another paternal allele missense mutation c.2654G>A (p.R885Q) in the exon 20 of the DUOX2 gene, which has been reported previously (3). No mutations in the TPO and TSHR genes were detected in this study. None of the controls showed the same pathogenic variants.

 The splicing site variant c.554+5C>T at the exon 4/intron 4 junction of the DUOXA2 was not present in the Human Gene Mutation Database, nor in the dbSNP database, 1000 Genomes Project database, or PubMed. The splicing variant prediction was carried out using Human Splicing Finder, Alternative Splice Site Predictor, and SplicePort. The prediction results showed that the variant might alter gene splicing by removing the normal splice donor at the abnormal site (potential splice site: ATGgtaagc, consensus value: 92.12) or (constitutive donor: TAAAGTTCCTgtaagtatta, score: 13.255; constitutive acceptor: tgtctcccagGAATCTCCCT, score: 9.038) or (donor short sequence: ttcctgtaagta, score: 1.59992; donor short sequence: ttcctgtattaa, score: -0.995081), respectively. We concluded that the c.554+5C>T might lead to intron 4 splicing loss and altered DUOXA2 messenger ribonucleic acid (RNA) sequence and the protein primary structure.

## DISCUSSION

The present study demonstrated compound heterozygous mutations, c.554+5C>T in the DUOXA2 gene and c.2654G>A (p.R885Q) in the DUOX2 gene in a pedigree with one four-year-old boy with GCH. H2O2 is a key element in iodine organification. DUOXA2/DUOX2 is the main enzyme for the H2O2-generating system. Defects in the DUOX2/DUOXA2 heterodimer lead to hypothyroidism and goiter. Since the first report in 2002 of DUOX2 mutations causing CH (10,11), over 40 mutations in the DUOX2 gene have been described correlated with CH, while only four mutations have been identified in the DUOXA2 gene (3,8,9,10). Thus far, our splice site mutation, as far as we know, is identified for the first time as being causative of CH.

The patients with DUOX2 or DUOXA2 mutation show a great genotype-phenotype variability (10,11). Maruo et al (3) firstly reported the p.R885Q mutation in the DUOX2 gene exhibiting transient hypothyroidism, which is not similar to our patient. The patient in this study had permanent CH and needed L-thyroxine replacement therapy. Heterozygous DUOX2 gene mutations result in different phenotypes, such as transient CH, subclinical hypothyroidism, and euthyroidism. However, the coexistence of heterozygous TSHR and DUOXA2 mutations causes overt hypothyroid condition (12).

Four mutations in the DUOXA2 (p.I26M, p.Y138X, p.C189R and p.Y246X) were found to be associated with CH (3,4,8,9,10). The patient with the p.I26M, p.C189R, and p.Y138X heterozygous missense mutation in DUOXA2 gene presented as a mild transient CH case. A homozygous nonsense mutation (p.Y246X) in patients with mild permanent CH and goiter was also identified. These patients are all of Chinese origin, indicating that this specific variant may occur at a high frequency in Chinese cohorts with CH.

Splice-site mutations are important disease-causing defects. It is estimated that approximately 10% of human genetic diseases are caused by mutations at splice sites (13). Analysis of the c.554+5C>T variation in the DUOXA2 gene revealed that it is capable of causing disease. Possibly this is the first report of a c.554+5C>T mutation in the DUOXA2 gene. The proband in this study presented with a normally located but enlarged thyroid gland. His parents, each with a single heterozygous mutation, both exhibited normal thyroid positioning and normal serum thyroid hormone levels. Our patient demonstrated no physical or cognitive developmental defects, primarily due to the timely and effective treatment.

Additionally, the c.554+5C>T mutation may affect the RNA transcription process and lead to genetic instability of the DUOXA2 gene. Further comprehensive functional assessments of the detected mutation will reveal its exact mechanism in the pathogenesis of CH. The R434X mutation in the DUOXA2 was detected by a two-stage strategy of genetic linkage studies and targeted sequencing of the candidate genes, suggesting a new testing strategy which uses next-generation sequencing in CH cases (14).

In conclusion, the present study reports a novel splicing site variant (c.554+5C>T) in the DUOXA2 gene and another missense mutation c.2654G>A (p.R885Q) in the DUOX2 gene. The findings indicate the importance of molecular genetic studies for the accurate diagnosis and classification of CH.

## ACKNOWLEDGMENTS

We thank the patient and his family members who agreed to participate in this study.

**Ethics**

Ethics Committee Approval: Huai’an Second People’s Hospital Ethics Committee (Approval number: 05-23-2014), Informed Consent: It was taken.

Peer-review: External peer-reviewed.

## AUTHORSHIP CONTRIBUTIONS

Concept: Shao-Gang Ma, Design: Shao-Gang Ma, Data Collection or Processing: Xiao Zheng, Ya-Li Qiu, Analysis or Interpretation: Man-Li Guo, Xiao-Juan Shao, Literature Search: Man-Li Guo, and Xiao-Juan Shao, Writing: Xiao Zheng and Shao-Gang Ma.

Financial Disclosure: The authors declared that this study was supported by the Social Development Project of Huai’an City (grant number: HAS2014005) and the Social Development Project of Suqian City (grant number: Z201460).

## Figures and Tables

**Table 1:**
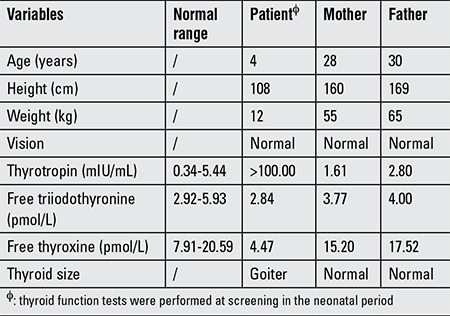
Clinical and biochemical data of the family in May 2015
